# Zygospore formation in Zygnematophyceae predates several land plant traits

**DOI:** 10.1098/rstb.2023.0356

**Published:** 2024-09-30

**Authors:** Charlotte Permann, Andreas Holzinger

**Affiliations:** ^1^ Department of Botany, University of Innsbruck, Sternwartestraße 15, 6020 Innsbruck, Austria

**Keywords:** cell wall, helicoidal pattern, lipid droplets, sexual reproduction, starch granules, zygospores

## Abstract

Recent research on a special type of sexual reproduction and zygospore formation in Zygnematophyceae, the sister group of land plants, is summarized. Within this group, gamete fusion occurs by conjugation. Zygospore development in *Mougeotia, Spirogyra* and *Zygnema* is highlighted, which has recently been studied using Raman spectroscopy, allowing chemical imaging and detection of changes in starch and lipid accumulation. Three-dimensional reconstructions after serial block-face scanning electron microscopy (SBF-SEM) or focused ion beam SEM (FIB-SEM) made it possible to visualize and quantify cell wall and organelle changes during zygospore development. The zygospore walls undergo strong modifications starting from uniform thin cell walls to a multilayered structure. The mature cell wall is composed of a cellulosic endospore and exospore and a central mesospore built up by aromatic compounds. In *Spirogyra*, the exospore and endospore consist of thick layers of helicoidally arranged cellulose fibrils, which are otherwise only known from stone cells of land plants. While starch is degraded during maturation, providing building blocks for cell wall formation, lipid droplets accumulate and fill large parts of the ripe zygospores, similar to spores and seeds of land plants. Overall, data show similarities between streptophyte algae and embryophytes, suggesting that the genetic toolkit for many land plant traits already existed in their shared algal ancestor.

This article is part of the theme issue ‘The evolution of plant metabolism’.

## 1. Introduction

**Figure 1.**
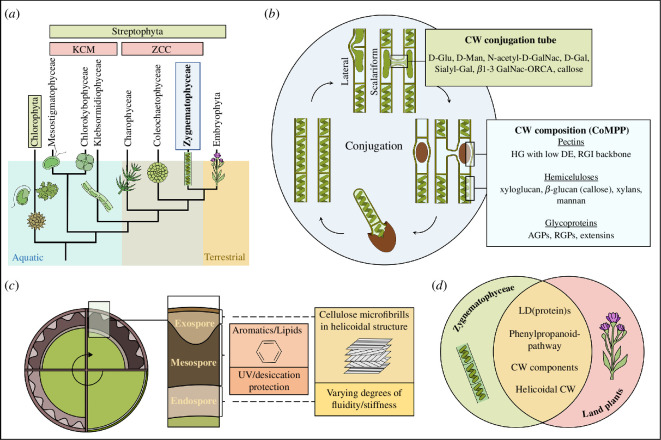
(*a*) Schematic representation of the phylogenetic position of Zygnematophyceae as sister to land plants based on Jiao *et al.* [1]. (*b*) Conjugation occurs in Zygnematophyceae as either lateral (within one filament) or scalariform (two filaments mate) (localized sugar residues after Kim *et al*. [2], Yoon *et al*. [3], Ikegaya *et al*. [4]; cell wall components detected by comprehensive polymer profiling (CoMPP) of zygospore-containing samples after Permann and colleagues [5,6] ‘rhamnogalactan-proteins’ (RGPs) as suggested by Pfeifer *et al*. [7]), (*c*) Schematic representation of zygospore ripening and possible composition of endospore, mesospore and exospore (based on [5,8,9]), (*d*) Overlapping metabolites, metabolic pathways and cell wall components between Zygnematophyceae and land plants.

About 470–450 Ma, descendants of streptophyte green algae began to occupy terrestrial habitats, leading to the evolution of land plants (embryophytes, figure 1*a*; reviewed in [10–14]). This process, termed ‘terrestrialization’, represents a fundamental event in Earth’s history and paved the way for life as we know it today. Land plants dominate the entire terrestrial macroflora as the only phototrophic lineage, which was able to rise above the substrate [15]. The evolution of highly organized vascular plants from streptophyte algae has long been speculated to involve a gradual increase in body complexity. However, recent phylogenetic studies have established the algal class Zygnematophyceae, which contains filamentous and unicellular forms, as sister lineage to land plants [15–18]. Plant evolutionary biology has, therefore, focused on the study of Zygnematophyceae and their possible similarities to land plants. These investigations will help uncover the key features of their common algal ancestor. Considerable efforts have resulted in the recent publication of the first zygnematophycean genomes (*Closterium* sp. [19], *Mesotaenium endlicherianum* and *Spirogloea muscicola* [20], *Penium margaritaceum* [1], *Zygnema circumcariantum* and *Zygnema cylindricum* [21]).

**Figure 2.**
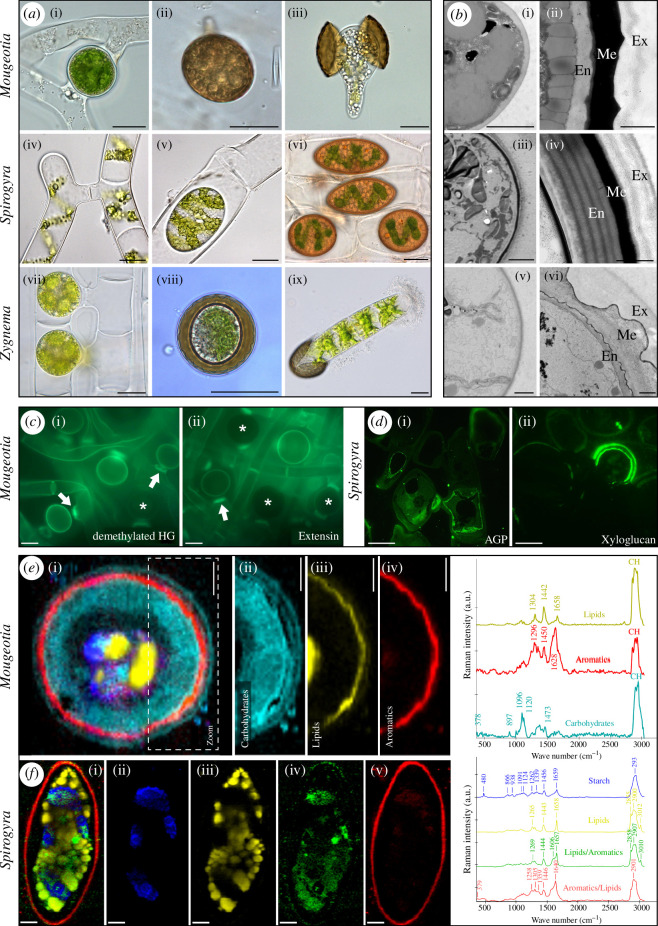
(*a*) Light micrographs of the conjugation process and zygospore formation in *Mougeotia*: extra gametangial scalariform conjugation (i), mature (ii) and germinating zygospore (iii) from Permann *et al*. [6]; *Spirogyra*: scalariform conjugation (iv), lateral conjugation (v) and mature zygospore (vi) from Permann *et al.* [5]; and *Zygnema*: young zygospores (vii), mature (viii) and germinating zygospore (ix) from Permann *et al.* [9], (*b*) Transmission electron micrographs of young (i,iii,v) and mature zygospores (ii,iv,vi) of *Mougeotia* [5], *Spirogyra* [5] and *Zygnema* [9], (*c*) Fluorescence microscopic staining of *Mougeotia* illustrating demethylated homogalacturonan (HG) and extensin (LM19; [6]), (*d*) Fluorescence microscopic staining of AGP (JIM13) and xyloglucan (LM15) in *Spirogyra* [5]. (*e*) Raman spectroscopy of *Mougeotia disjuncta* with respective spectra of carbohydrates, lipids and aromatics [6]. (*f*) Raman spectroscopy of *Spirogyra* sp. with respective spectra of starch, lipids, lipids/aromatics, aromatics/lipids [31]. All images are reproduced with permission (CC-BY). Scale bars: (*a,c,d*) 20 µm, (*b*(i)) 2.5 µm (ii) 1 µm, (*e*) 5 µm, (*f*) 6 µm.

Zygnematophyceae are found worldwide in a variety of environments. Their habitats often exhibit semi-terrestrial conditions, which exposes them to increased abiotic stresses. Their vegetative stress tolerance has been extensively studied and the results are summarized in several review articles [22–24]. However, another trait, sexual reproduction, is crucial for the persistence of present-day streptophyte green algae in semi-terrestrial habitats. Sexual reproduction most probably also played a role in the transition of their ancestral taxon to land. The development of a spore-producing phase in the life cycle of plants as adaptation to terrestrial conditions was crucial in the evolution of early land plants [25]. A detailed revision of the evolution of life cycles, meiosis and sexual reproduction in plants and algae with a focus on the transition from unicellular organisms to multicellularity is provided by Niklas *et al*. [26]. While land plants exhibit a heteromorphic life cycle and streptophyte algae are unicellular or filamentous, each life cycle involves gamete fusion, meiosis and an alternation between a haploid and a diploid phase [10,26]. In streptophyte algae, sexual reproduction has not been described in members of the KCM-grade (Klebsormidiophyceae, Chlorokybophyceae and Mesostigmatophyceae; according to de Vries *et al*. [27]; figure 1*a*), whereas it is well known from the later-diverging Zygnematophyceae [28], Coleochaetophyceae [29] and Charophyceae (figure 1*a*; [30] ZCC-grade according to de Vries *et al*. [27]). Only in Zygnematophyceae, sexual reproduction occurs by conjugation, resulting in the formation of zygospores (figures 1*b* and 2*a*). The phylogenetic position of this algal class indicates that these taxa may convey important information about the properties of the last common ancestor of streptophyte algae and land plants. While in both lineages independently derived evolution has taken place over time, conjugation and the characteristics of the resistant zygospores (figure 1*c*) might represent a key adaptation strategy in the process of terrestrialization.

Leaving the aquatic environment and colonizing land is associated with a significant increase in abiotic stresses such as desiccation, high UV and photosynthetic irradiance and more rapid temperature shifts [22–24]. The synthesis of specialized cell walls with a specific composition is a key prerequisite for thriving in these highly stressful environments and for the evolution of land plants [32]. This important feature is also found in the zygospores of Zygnematophyceae, which exhibit a multilayered cell wall structure (figure 2*b*). The zygospore wall entails many components (figure 1*c*) and ultrastructural arrangements (figure 1*c*) not found in the vegetative state, but similar to spores of bryophytes and ferns and pollen of seed plants (figure 1*d*). These findings and other similarities between Zygnematophyceae and land plants suggest that many key traits and metabolites that have been critical for their unprecedented success in terrestrial habitats have been inherited from their common algal ancestors.

In this review, we summarize the current knowledge on the special type of sexual reproduction and zygospore formation occurring in Zygnematophyceae, the sister lineage to land plants, and discuss their metabolic and structural similarities in the context of terrestrialization and plant evolution.

## 2. Conjugation in Zygnematophyceae

Sexual reproduction by conjugation is a homoplastic trait that evolved in Zygnematophyceae and zygomycetous fungi [33,34]. The process of conjugation in Zygnematophyceae involves the transformation of the vegetative cell contents into morphologically indistinguishable gametes. While being an isogamous organism, male and female terms are assigned to the gametangia based on gamete migration (gamete of the male ‘donor’ cell migrates to the female ‘recipient’ cell). The formed gametes do not possess locomotory organelles such as flagella [33]. One hypothesis suggests that sexual reproduction by conjugation lacking flagellated gametes is more effective and evolved as adaptation to glacial habitats during the Cryogenian Snowball Earth [34,35]. Fusion of the gametes, located inside the gametangia, is facilitated by a conjugation tube and does not require the availability of water, which is particularly advantageous in semi-terrestrial habitats [36].

There are different modes of conjugation depending on the origin of the conjugation tube and the site of gamete fusion, which is an important taxonomic criterion (figure 1*b*). Scalariform conjugation is the process of two different filaments mating, while in lateral conjugation two neighbouring cells within one filament conjugate (figure 1*b*). The formed zygospores are diverse in form, colour and surface structure. A detailed summary of conjugation features in Zygnematophyceae is provided by the comprehensive species guide of Kadlubowska [28]. Conjugation and zygospore characters are crucial in traditional species determination. However, it should be mentioned that studies on the genus *Zygnema* revealed a difference between existing taxonomic concepts of classification and molecular phylogeny [37]. As conjugation is often subject to seasonality and difficult to observe in the field or rare in some habitats [38,39], links between morphologically assigned species and molecular sequences are generally difficult to obtain.

### (a) Induction of conjugation

Successful induction of conjugation in Zygnematophyceae under laboratory conditions has been reported only occasionally (e.g. [4,5,7,40–43]. Most studies have been performed on the genus *Spirogyra*, while only a few reports of induced conjugation are available for *Zygnema* [40] and *Mougeotia* [44]. Cultures of *Spirogyra* sp. have been shown to conjugate when placed in a nutrient-poor medium and exposed to a higher light intensity than used under standard culture conditions [5,42,43]. In an early study on *Sirogonium*, the application of high light intensity was also inductive to sexual reproduction [45]. However, an inhibiting effect on sexual reproduction has been reported for UV radiation [41,42]. Analysis of conjugation processes in *Spirogyra* has recently led to the identification of 13 *Spirogyra* spp. based on sexual reproduction traits induced under laboratory conditions [43]. While these results suggest high light intensity and nitrogen depletion as key factors for the induction of conjugation in Zygnematophyceae, the necessary settings and triggers are not fully understood and consistently conjugating laboratory cultures are rare. It has been speculated that seasonality and internal factors may also be involved in the onset of conjugation. This was suspected in *Spirogyra mirabilis*, where successful induction of conjugation was only observed in early spring [5]. Kim & Kim [46] also hypothesized that some field-collected cultures lose their ability to conjugate as a result of adaptation to laboratory conditions.

### (b) Genes involved in conjugation

There are a limited number of studies dealing with the genes involved in the process of sexual reproduction in Zygnematophyceae. Recently, a reversion from anisogamy to isogamy has been proposed for Zygnematophyceae [19]. This is in contrast to the other members of the ZCC-grade that exhibit anisogamy. Isogamous sexual reproduction involves genetic differences between the gametes and thus different mating types (MTs), resulting in offspring with novel gene combinations. Older studies also described a heterothallic nature in *Zygnema circumcarinatum*, and genetic backcrossing proposed four distinct classes of mating types [40,47,48]. In his studies, Miller [48] proposed that heterothallism in this alga is determined by two or more loci located on the same chromosome. More recent data on the genetic background of sexual reproduction in Zygnematophyceae conducted on *Closterium* sp. suggest that a single genetic locus is responsible for MT (mt+ and mt-) determination in this genus [49,50]. A gene (*CpMinus1*) associated with the mt- phenotype has been proposed as the major MT determinant [19]. Biochemical studies have also shown that different sex pheromones are released by MTs towards each other [51]. It is hypothesized that isogamous sexual reproduction in Zygnematophyceae evolved after their divergence from the land plant lineage and that sperm-related genes were lost in Zygnematophyceae, which is consistent with the loss of flagellated gametes. Isogamous sexual reproduction, including the *CpMinus1* gene, therefore, most probably arose independently in Zygnematophyceae [19]. While these studies provide valuable new insights, further research is needed on the mode of MT recognition, MT inheritance and physiological segregation of MT in Zygnematophyceae.

### (c) Cell wall composition during conjugation

The composition of vegetative cell walls of streptophyte algae is manifold and differs between early and late divergent taxa [52]. Taxa of the later-diverging ZCC-grade are similar in their cell wall composition to land plants, including polysaccharides like cellulose (β-(1-4) glucan), pectins (homogalacturonans and rhamnogalacturonan-I), callose (β-(1-3) glucan), hemicelluloses (xyloglucans, mannans and xylans; [6,52]) and arabinogalactan-proteins [7]. Zygnematophyceae are, moreover, often covered by a sticky mucilage layer. This extracellular matrix is mainly composed of acidic polysaccharides, including homogalacturonans (polymers of galacturonic acid) and arabinogalactan proteins (AGPs, with polymers of galactose and arabinose [1,53–55]).

Cell walls are dynamic constructs that react to biotic or abiotic stress as well as developmental processes by changing their structure and composition [56]. While sexual reproduction represents an important developmental stage in the life cycle of Zygnematophyceae only very few reports on the cell wall components involved are available. In *Penium margaritaceum*, a conjugation process with six different stages was described (but only by the rather nonspecific Alcian blue staining) and the possible involvement of mucilage in cell adhesion and cell–cell communication was suggested [57]. A little more detail was provided by Kim *et al*. [2], who used different lectins labelled with fluorescein isothocyanate to monitor the conjugation process in *Zygnema cruciatum*. Kim *et al*. [2] reported that Concavalin A (Con A; specific for D-glucose and D-mannose) and *Rhicinus communis* agglutinin (RCA; specific for D-galactose) did not label the vegetative filaments, but were found on swollen papillae (table 1); some glycoconjugates with N-acetyl-D-galactosamine residues appeared at the tip of the male papillae and later on the female papillae. Blocking experiments with the complementary lectins soybean agglutinin (SBA) and/or peanut agglutinin (PNA) showed that the secreted materials might be involved in signalling or recognition between male and female papillae [2]. Ikegaya *et al*. [4] characterized the conjugation process using lectins and found conjugation tubes stained with *Bandeiraea (Griffonia) simplicifolia* lectin (BSL-I) specific for D-galactose > N-acetyl-D-galactosamine and jacalin (lectin isolated from jackfruit *Artocarpus integrifolia* seeds) specific for D-galactose (table 1). Studies on the conjugation process in *Spirogyra varians* showed that Con A, RCA and *Ulex europaeus* agglutinin (UEA; α-D-fucose) bind to the extracellular materials during the conjugation process [3]. Con A was furthermore shown to label the papillae of conjugating *Closterium* sp. [58]. From these studies, it can be concluded that the conjugation papillae contain polysaccharides either with a terminal N-acetyl-D-galactosamine or a D-galactose residue. It should be noted that these results are not congruent between the different genera studied, and in some cases are even contradictory, possibly because, e.g. *Zygnema* and *Spirogyra* papillae contain different residues (table 1). In addition, these studies do not allow a firm conclusion on the composition of polysaccharides (or oligosaccharides). The copulation papillae are highly dynamic and a constant turnover is likely, so that N-glycosylated enzymes could be available in the cell walls (i.e. glucanases, glucosidase, transglycanases and transmembrane receptors).

**Table 1.** Comparison of lectin binding in different species of conjugating Zygnematophyceae. CoMPP indicates that the binding has been detected by comprehensive microarray polymer profiling in samples containing conjugating filaments and zygospores, but no localization was possible [5].

lectin	specification	species	vegetative filament	conjugating papillae	reference
concavalin A (Con A)	D-glucose, D-mannose	*Zygnema cruciatum*	negative	positive	[2]
		*Spirogyra varians*	positive	positive	[3]
		*Spirogyra* sp.	negative	positive	[4]
		*Closterium* sp.	positive	positive	[58]
B-1005	a-linked mannose	*Spirogyra mirabilis*	CoMPP	CoMPP	[5]
*Ulex europaeus* agglutinin (UEA)	L-fucose	*Zygnema cruciatum*	positive	negative	[2]
		*Spirogyra varians*	negative	positive	[3]
B-1065	a-linked-fucose	*Spirogyra mirabilis*	CoMPP	CoMPP	[5]
soybean agglutinin (SBA)	N-acetyl-D-galactosamine	*Zygnema cruciatum*	negative	positive	[2]
		*Spirogyra varians*	negative	negative	[3]
		*Spirogyra* sp.	negative	positive	[4]
peanut agglutinin (PNA)	D-galactose, N-acetyl-D galactosamine	*Zygnema cruciatum*	negative	positive	[2]
		*Spirogyra varians*	negative	negative	[3]
*Ricinus communis* agglutinin (RCA)	D-galactose	*Zygnema cruciatum*	negative	occasional	[2]
		*Spirogyra varians*	negative	positive	[3]
(RCA-I)		*Spirogyra* sp.	negative	positive	[4]
B-1085	Gal and GalNAc residues	*Spirogyra mirabilis*	CoMPP	CoMPP	[5]
*Dolichos biflorus* agglutinin (DBA)	N-acetyl-D-galactosamine	*Zygnema cruciatum*	negative	negative	[2]
		*Spirogyra varians*	negative	negative	[3]
wheat germ agglutinin (WGA)	neuramic acid	*Zygnema cruciatum*	negative	negative	[2]
		*Spirogyra varians*	negative	negative	[3]
		*Spirogyra* sp.	negative	negative	[4]
BSL-I	D-galactose, N-acetyl-D-galactosamine	*Spirogyra* sp.	negative	positive	[4]
B1405	Gal and GalNAc	*Spirogyra mirabilis*	CoMPP	CoMPP	[5]
jacalin	Sialyl-gal, β1 – 3 GalNAc-ORCA	*Spirogyra* sp.	negative	positive	[4]

## 3. Zygospore cell wall composition shows similarities to land plants

### (a) Cell wall composition in zygospore-containing samples analysed by comprehensive microarray polymer profiling

Recently, comprehensive microarray polymer profiling (CoMPP) was performed in zygospore-containing samples of *Mougeotia disjuncta* [6] and *Spirogyra mirabilis* [5], allowing the simultaneous testing for the presence of up to 46 cell wall epitopes. This method includes a two-step extraction of cell wall components with cyclohexane diamine tetraacetic acid (CDTA) and NaOH. CDTA chelates Ca^2+^ ions and is thus an excellent solvent for Ca^2+^ cross-linked pectins, while NaOH is used as subsequent solvent for hemicellulose-rich polymers [59].

In *S. mirabilis*, CDTA extracted high amounts of homogalacturonan (HG), with LM19 antibody (binding to HG with low degree of esterification (DE)) showing the strongest signal, followed by JIM5 and 2F4 antibodies, which recognize similar epitopes [5]. Similarly, in *M. disjuncta*, JIM5 and LM19 showed the strongest binding signals of all epitopes in the CDTA fraction, suggesting that HG with low DE were dominant in this sample [6]. In addition, binding of LM20 (HG with higher DE) and INRA-RU1 + 2 (rhamnogalacturonan I (RGI)) was observed [6]. Interestingly, some probes (such as INRA-RU1) produced a similarly strong signal in the CDTA and the NaOH fractions, suggesting that the respective epitopes are tightly integrated into the cell wall and/or are part of different cell wall domains. Binding was also found for LM16, which recognizes an arabinose-rich side chain of RGI [6]. The second major class of cell wall components in the CDTA fraction of *S. mirabilis* and *M. disjuncta* were glycoproteins, yielding high binding of antibodies recognizing different motifs on arabinogalactan proteins (AGPs; JIM13, MAC207, LM14) and extensins (JIM11, LM3, JIM20, LM1, JIM13; [5,6]). The existence of *Spirogyra pratensis* arabinogalactan proteins was recently confirmed by Pfeifer *et al*. [7]. These authors showed a strong reactivity of a *S. pratensis* Yariv fraction with the JIM13 antibody and performed a detailed analysis of the polysaccharide fractions leading to the structural proposal of a ‘rhamnoglactan-protein’ [7].

The NaOH fraction of *S. mirabilis* was dominated by β-glycans, especially mannans, with antibodies against linear mannans (BS-400-4) and hetero-mannans (LM21) showing the strongest signals. In contrast, binding to mannans was less abundant in *M. disjuncta* [6]. In *S. mirabilis* and *M. disjuncta,* abundant epitopes of xyloglucans (LM15, LM25), xylans (LM10, LM11) and callose (BS-400-2) were found [5,6]. While in *M. disjuncta* no binding with lectin epitopes was found, in *S. mirabilis* some lectin probes showed binding in the CDTA fraction and recognized mannose (B-1005), fucose (B1065), galactose and N-acetylgalactosamine residues (B-1085, B-1405; table 1; [5]). These sugars have also been observed and differentially localized in vegetative and conjugating filaments of *S. varians* [3] and *Spirogyra* sp. ([4]; table 1).

### (b) Localization of cell wall epitopes in conjugating filaments and zygospores

To date, limited data are available on the localization of cell wall polymers in zygnematophycean zygospores. A comprehensive study was performed in *M. disjuncta*, where *in situ* staining with probes for the most abundant polymers was performed based on CoMPP analysis [6]. Homgalacturonans with low DE were probed with a more recently developed LM19 antibody in *M. disjuncta*, showing abundant probe binding to algal filaments, especially in the conjugation tubes and suspensor regions (figure 2*c*; LM19). However, only about 50% of the present zygospores were stained with this probe, suggesting a developmentally dependent appearance of this epitope [6]. In a complementary approach to the LM19 antibody staining, another very small HG probe (OG7 − 13AF488) that binds to de-methylated HG was used and gave similar results (figure 2*c*; OG7-13AF 488). However, even with this small probe no staining of the inner cell wall layers was observed [6]. To further test the specificity of this probe, cells were digested with pectate lyase, which stained only cell wall remnants [6]. The INRA-RU1 (rhamnogalacturonan I (RGI)) specific antibody resulted in binding in (i) an outer zygospore sheath, suggesting the presence of fibrous RGI structures and in (ii) highly localized spots within the sheath of a zygospore [6]. Treatment of zygospores with CDTA prior to staining removed the INRA-RU1 epitopes from the surface, but the highly localized spots inside the zygospores remained, suggesting that they represent RGI secretion hotspots [6]. Antibodies against callose (400-2) stained the suspensor region, antibodies against xyloglucan (LM25) and extensin (JIM20) stained the cross walls of filaments, while JIM13 (AGP) stained the outer zygospore wall in *M. disjuncta* [6]. In semi-thin sections of *S. mirabilis* zygospores stained with JIM13 (AGP) antibody, the cell walls of a conjugating filament were stained (figure 2*d*; JIM13; [5]). A xyloglucan-specific antibody (LM15) showed a bilayer arrangement of staining, probably representing the exospore and endospore of the zygospore (figure 2*d*; LM15; [5]). Pfeifer *et al*. [7] showed distinct staining in *Spirogyra* sp. with β-glucosyl Yariv’s reagent (βGlcY) within the donor and receptor cells, while the cell walls of the zygospores were not directly stained.

## 4. Zygospore development is accompanied by rearrangement processes

### (a) Rearrangement of organelles and storage compounds during zygospore maturation

**Figure 3.**
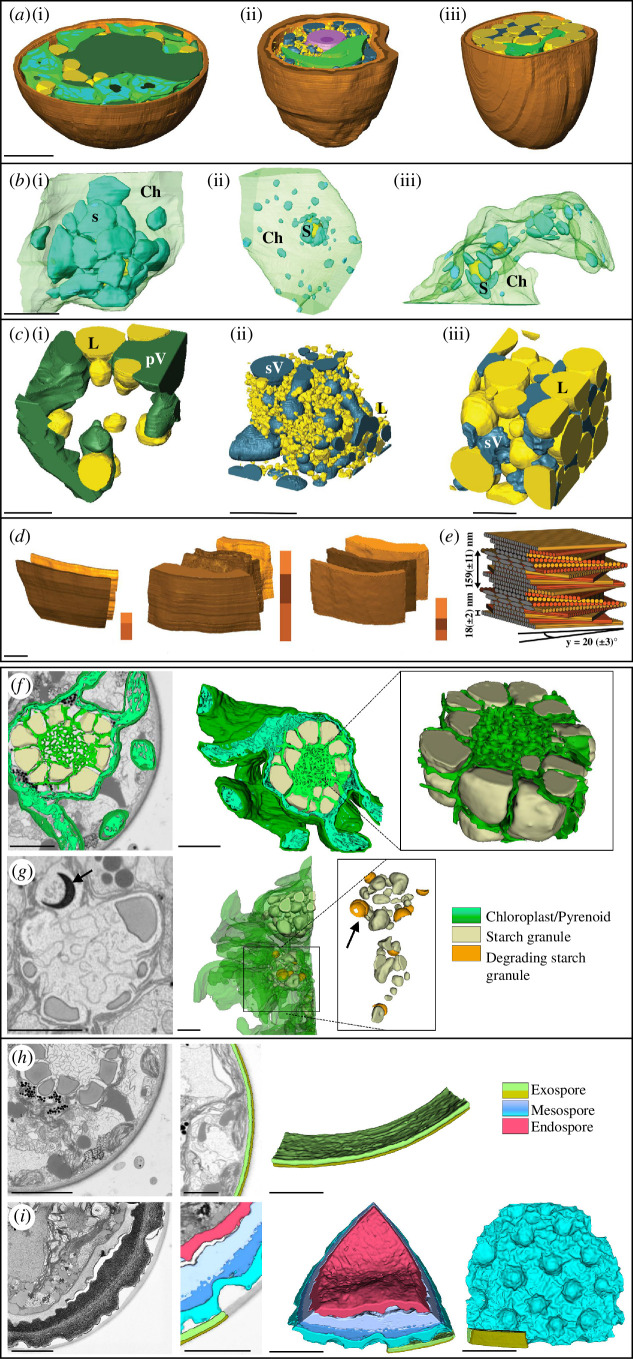
Three-dimensional reconstruction of zygospores ((i), young; (ii, iii), mature) in *Spirogyra* ((*a–e*), from Antreich *et al*. [60], figs. 2–4) and *Zygnema vaginatum* ((*f–i*), from Permann *et al*. [9], figs. 6, 7). (*a*) Overview, (*b*) chloroplasts and starch granules, (*c*) lipids and vacuoles, (*d*) cell wall layers, (*e*) three-dimensional reconstruction of the helicoidal pattern of the cellulose microfibrils, (*f*) chloroplast and pyrenoid with starch grains in young zygospore, (*g*) degrading starch grains (arrows) in mature zygospore, (*h*) cell wall in young zygospore, (*i*) cell wall in mature zygospore. All images are reproduced with permission (CC-BY). Abbreviations: Ch, chloroplast; L, lipid; pV, primary vacuole; S, starch; sV, secondary vacuole. Scale bars: (*a*) 10 µm, (*b,c,f–i*) 5 µm, (*d*) 2 µm.

The maturation process of zygospores was described by light and transmission electron microscopy in *Mougeotia parvula* and *M. disjuncta*, suggesting that zygospores accumulate storage compounds in the form of ‘lipid droplets’ (figure 2*b*; [6]). Further analysis by Raman spectroscopy in *M. disjuncta* detected lipids in the centre of the zygospore, but also as a continuous layer surrounding the cell, with an endmember spectrum showing bands at 1658, 1442 and 1304 cm^−1^ (figure 2*e*; [6]). Older *Mougeotia* zygospores contained massive accumulations of lipid droplets, either throughout the zygospore or at the periphery closely associated with the zygospore wall (figure 2*b*; [6]). A similar observation was made in zygospores of *S. mirabilis*, where lipid droplets were observed in the lumen of mature zygospores [5]. Their biochemical nature was confirmed as lipids by Raman spectroscopy with a spectral signature of the lipidic component similar to linoleic acid (figure 2*f*; [31]). However, it has not been possible to quantify the changes and rearrangements of storage compounds in zygospores. Significant new observations on the arrangement of storage compounds were accomplished after the three-dimensional reconstruction of different *Spirogyra* sp. zygospores after serial block-face scanning electron microscopy (SBF-SEM; figure 3*a–c* [60]) and *Zygnema vaginatum* zygospores after focused ion beam scanning electron microscopy (FIB-SEM; figure 3*f*,*g*; [9]).

In *Spirogyra* sp., a high variability in lipid droplet size and abundance was observed during zygospore maturation [60]. In young zygospores of *Spirogyra* sp. ‘Greece’, the lipid droplets were large (21 ± 18 μm³; figure 3*c*(i)), whereas in the mature zygospores of *Spirogyra* sp. ‘Tyrol’ and ‘Greece’ the numerous lipid droplets were much smaller (0.1 ± 0.2 and 0.5 ± 0.9 μm³; figure 3*c*(ii)). In contrast, in mature zygospores of *S. mirabilis*, the lipid droplets were significantly larger (34 ± 29 μm³; figure 3*c*(iii)) and occupied about half of the zygospore lumen, suggesting a significant total increase in lipid volume [60]. A reorganization of lipid droplets during zygospore maturation has also been reported in *Zygnema vaginatum* [9]. While in the cell lumen of young zygospores, multiple lipid droplets of different shapes and sizes (average volume of 1.83 ± 3.03 μm^3^ (*n* = 63) and a surface/volume ratio of 7.23 ± 2.58) were detected, lipid droplets at the intermediate developmental stage of zygospore maturation were spherical in shape (average volume 0.49 ± 1.21 μm^3^; surface/volume ratio 9.04 ± 2.52) and sometimes found in close contact with the innermost layer of the cell wall [9]. Mature zygospores showed a layer-like distribution of lipid droplets that began to coalesce into a large cluster, making detailed size measurements impossible [9]. In young zygospores of *Z. vaginatum*, lipid droplets accounted for approximately 11.77% of the total cell volume, while this percentage increased to 19.45% in the mature zygospore [9]. In *Z. vaginatum,* lipid droplets were found in direct contact with network-forming mitochondria that play an important role in the glyoxylate cycle; however, peroxisomes were not segmented in our FIB-SEM analysis [9]. We have, therefore, only indirect indication of a possible metabolic utilization of fatty acids from triacylglycerol stored in lipid bodies via β-oxidation, which is performed in peroxisomes [9].

In *Z. vaginatum*, a degradation of chloroplasts during the maturation process leads to a reorganization of the internal zygospore structure [9]. In young zygospores, the lobed chloroplasts were well developed and contained pyrenoids (figure 3*f*). The pyrenoids were surrounded by multiple starch granules and penetrated by a dense network of intrapyrenoidal membranes in a gyroid-like appearance [9]. Chloroplasts and pyrenoids at an intermediate stage of development showed a reduced density of the intrapyrenoidal membranes as well as ‘crescent-shaped’ degrading starch granules (figure 3*g*). In contrast, highly degraded pyrenoids surrounded by only a few starch granules and remnants of the intrapyrenoid membranes occurred in mature *Z. vaginatum* zygospores [9]. Comparisons of the starch granule size between the developmental stages revealed a mean volume of 3.58 ± 2.35 μm^3^ with a surface/volume ratio of 4.47 ± 2.11 (*n* = 70) in young cells and approximately 0.68 ± 0.74 μm^3^ and 8.06 ± 3.34 (*n* = 83), respectively, in intermediate cells. In mature zygospores, the degree of starch granule degradation was too high for this analysis [9]. Similar observations were made in *Spirogyra* sp. during the maturation process (figure 3*b*). Starch degradation was quantified by the three-dimensional reconstructions, where starch granules in young *Spirogyra* sp. ‘Greece’ had a significantly (*p* < 0.05) higher volume of 8 ± 5 μm³ when compared with mature zygospores of the same strain (0.2 ± 0.2 μm³) and *S. mirabilis* (0.6 ± 0.6 μm³) or *Spirogyra* sp. ‘Tyrol’ (0.3 ± 0.2 μm³), which were not significantly different [60]. The reduction in starch granule size in young versus older *Spirogyra* sp. zygospores is illustrated in figure 3*b* [60]. Raman analysis confirmed the presence of the starch granules (marker bands 477, 936, 1121, 1350 and 2906 cm^−1^) around the pyrenoids [31,60]. Early ultrastructural investigations conducted on *Spirogyra* sp. also confirm accumulations of starch granules in the cell lumen of zygospores [61].

### (b) Building up the complex zygospore wall

While the ultrastructural properties of the zygospore wall are a unique key feature of zygospores, only a few early transmission electron microscopy (TEM; e.g. [61,62–64]) and SEM studies (e.g. [65,66]) are available. In addition, more recent TEM data on *Mougeotia* [6], *Spirogyra* [5,31,60] and *Zygnema* [9,39,67] helped shed light on the complex internal zygospore wall architecture. In contrast to the single-layered nature of thin vegetative cell walls, the zygospore cell wall exhibits a multi-layered structure, including three main layers, termed endospore, mesospore and exospore (figure 2*b*). However, there are significant differences between the investigated genera, which will be highlighted in the following section along with their development.

In *Mougeotia*, young zygospores are surrounded by a uniform, thin and polysaccharidic zygospore wall (figure 2*a*(i), *b*(i) [6]). Upon maturation, the zygospore wall develops a dark brown mesospore (figure 2*a*), which exhibits an electron dense appearance in TEM micrographs (figure 2*b*(ii)). The Raman signature with bands at 1628, 1600, 1450 and 1296 cm^−1^ indicates an aromatic and lipidic nature of the mesospore in *M. disjuncta* (figure 2*e*). The observed high fluorescence in this layer and the sensitivity to sample degradation are typical for aromatic components, such as lignin [68] or sporopollenin [69]. The deposition of sporopollenin, a highly resistant biopolymer, in the coat of spores is one of the earliest traits to evolve in the ancestral Embryophytes and a key adaptation to terrestrial habitats [70]. Although a biochemical analysis of the mesospore composition in *Mougeotia* is lacking, we have several indications from Raman spectroscopic analysis that it contains sporopollenin-like material [5]. In *Mougeotia,* an additional fourth lipid-like layer, which was located between the endospore and mesospore, has been described [6]. The thin endospore and exospore contained various polysaccharides, which was consistent with their loose fibrillar appearance and our detection of carbohydrates in the wall (see §3b).

In *Spirogyra*, three main types of zygospores, differing in shape (ovoid, ellipsoidal and lenticular) and number of mesospore layers (single- or double-layered) have been described [43]. The mesospore is an important taxonomic feature and is mostly smooth in *Spirogyra*, only three species with a reticulated mesospore (*S. californica*, *S. australica* and *S. croasdaleae*) having been described [71]. The colour of the mesospore varies but is mostly yellow-brown to brown [71]. In TEM micrographs, the mesospore of *S. mirabilis* [5] and another *Spirogyra* sp. [31] have an electron dense appearance (figure 2*b*(iii,iv)). In a Raman fluorescence image of *S. mirabilis*, the innermost part of an open zygospore showed a strong intensity, indicating a high content of aromatic compounds [6]. The extracted zygospore cell wall spectrum shows clear aromatic band contributions at 1637 cm^−1^ with shoulders at 1604 and 1575 cm^−1^, suggesting a complex aromatic nature [6]. In order to gain insight into the components reflected in the cell wall spectrum, a greedy algorithm-based method, which stepwise updates the coefficients related to the contribution of each library (orthogonal matching pursuit (OMP)) was used. A *Lycopodium* spore cell-wall spectrum was selected by the algorithm to model the average spectrum of the *S. mirabilis* zygospore [5]. In addition to the *Lycopodium* spore cell-wall spectrum with main bands at 1604 and 1451 cm^−1^, flavanones and lipids were included in the fit to account for the bands at 1637, 1575 and 1296 cm^−1^. In a field sample of *Spirogyra* sp., a lipidic/aromatic component showed a distinct patterned appearance in the zygospore wall, but it was mostly accumulated in the centre of the zygospore (figure 2*f*, green spectrum; [31]). The aromatic/lipidic component, which was localized directly in the zygospore wall of *Spirogyra* sp., showed an additional cellulose band at 379 cm^−1^ (figure 2*f*, red spectrum; [31].

The endospore and exospore of different *Spirogyra* sp. were drastically different in sizes (figure 3*d*) and most interestingly showed a distinct internal arrangement of the microfibrils in a helicoidal pattern as seen in TEM micrographs (figure 2*b*(iv); [5,60]). Data from the analysis of HPF/FS zygospores of *Spirogyra* sp. allowed a detailed analysis of the helicoidal microfibril arrangement (figure 3*e* [60]). A similar symmetrical arrangement of multiple fibrillar layers has so far only been observed in the endospore of *Zygnemopsis lamellata* zygospores but was not discussed further [39]. Analysis of the pitch angle of the microfibril layers in the endospore was performed in different *Spirogyra* sp. These angles were determined to be approximately 18 ± 3° in *S. mirabilis*, 20 ± 3° in *Spirogyra* sp. ‘Tyrol’ and 38 ± 8° in *Spirogyra* sp. ‘Greece’ [60]. A three-dimensional reconstruction of the microfibril arrangement in *Spirogyra* sp. ‘Tyrol’ is shown in figure 3*e*.

In *Zygnema vaginatum*, particularly, massive zygospore walls were observed (figure 2*a*(vii-ix)) and their formation was visualized by three-dimensional reconstruction after FIB-SEM [9]. The exospore of young zygospores was small and had a uniform appearance in TEM micrographs (figure 2*b*(v)), the three-dimensional reconstruction allowed for distinguishing two layers (figure 3*h*; [9]). After zygospore maturation in older zygospores a massive mesospore is visible in TEM micrographs (figure 2*b*(vi)), which appears multi-layered in the three-dimensional reconstructions, giving the mesospore of *Z. vaginatum* the typical appearance with many indentations (figure 3*i*; [9]).

## 5. Similarities to land plants

The establishment of plants in terrestrial habitats required a number of prerequisites, as the transition from water to land was accompanied by an increase in abiotic stresses. It is most likely that parts of the environmental response toolbox used by the earliest land plants were already present in their algal ancestors and are, therefore, still present in Zygnemtophyceae today. As spores, seeds and pollen are an essential part of the life cycle of land plants, the sexually formed zygospores of Zygnematophyceae may contain shared metabolic traits or precursors of metabolites and provide insights into the state of their algal ancestors.

### (a) Specialized metabolite pathways

As described above, zygospores of Zygnematophyceae are characterized by a resilient cell wall rich in aromatics and accumulate lipids and starch as storage products. In particular, their middle mesospore has been suggested to provide effective protection against UV and desiccation stress [5,6,9,31]. In this context, a sporopollenin-like material has been proposed in several zygnematophycean zygospores [5,6,31,67,72,73] as well as in zygospores of other members of the ZCC-grade (Coleochaetophyceae [74]; Charophyceae [75]). Moreover, spectral similarities of *S. mirabilis* and *Lycopodium* spores have been reported (see §4b [6]). Sporopollenin is a non-hydrolysable macromolecule found in bryophyte spores and the coats of land plant pollen. The transfer of sporopollenin from charophycean zygospores to the spore walls of the haploid cells after meiosis is seen as a key factor in the evolution of land plants [25,70]. A remarkable conservation of biochemical pathways for sporopollenin biosynthesis across the plant kingdom leading to the polyhydroxylated polyketide-based subunits is suggested [76]. Polyketides are polyphenolic compounds that have their origin in the phenylpropanoid pathway [77], a pathway that has been crucial for the evolution of land plants as it encompasses precursors for thousands of specialized metabolites dealing with environmental stressors [25,78–82]. It has been shown that many key components of the genetic makeup for dynamic stress responses found in land plants are already present in streptophyte algae [83]. While it has long been thought that this pathway is unique to land plants and evolved within that lineage, parts of the phenylpropanoid pathway and derived metabolites have recently been reported in streptophyte algae [81,82,84]. These findings shed light on the evolutionary history of one of the defining traits of embryophytes and show that the genetic toolkit for specialized metabolism predates the evolution of land plants.

### (b) Complex internal microfibrillar orientation

The majority of land plants and algal species contain cellulose as major cell wall component. However, there are variations in the structure of their cellulose-synthesizing enzyme complexes (terminal complexes). While in chlorophyte algae cellulose is synthesized in linear terminal complexes, land plants and streptophyte algae share rosette terminal complexes [85–87]. The rosette terminal complexes have already been visualized in early works in *Micrasterias* [88] and *Spirogyra* [89]. The feature of cellulose-synthesizing complexes arranged in rosettes is thought to be a key factor in the evolution of land plants as it is linked to the origin of a complex body plan based on their influence on cytokinesis and intercellular communication [85,90]. Similarities of cellulose synthase (CesA) genes between land plants and streptophyte algae suggest that CesA domains, characteristic of land plants, arose before their evolutionary split [85]. The terminal complex structure is also hypothesized to affect cellulose microfibril size, shape, crystallinity and intramicrofibrillar associations [85,91]. These microfibril characters affect the flexibility and integrity of the cell wall and, therefore, also its function [92].

Ultrastructural studies on the internal microfibrillar structure of zygospore cell-wall layers revealed a helicoidal pattern in the endospore and exospore of *Z. lamellata* [39] and several *Spirogyra* strains [31,60]. Helicoidal patterns are well known from the secondary walls of stone cells of seed plants and are described as highly organized and flexible [8,93,94]. Further atomic force microscopy analysis confirmed this highly complex internal structure and its variability in stiffness [31]. Recently, a more detailed analysis of this microfibrillar arrangement, including pitch angle calculations, was performed in *Spirogyra* spp. [60]. The pitch angle and the thickness of the lamellae can modulate the mechanical properties of the cell wall layer at the nanoscale. In-depth studies of walnut shell cell walls provided critical and novel information on the morphogenesis and composition of such unique and robust structures in seed plants [95–97]. The internal microfibrillar orientation in a helical pattern is highly advantageous as its organized and flexible nature results in varying degrees of fluidity and stiffness, making it adaptable to changing physiological conditions of growth and specialization. In addition, this pattern and the presence of glucuronoxylan (GX, hemicellulose) are associated with cell wall lignification [8]. While no GX was detected in Zygnematophyceae, the hemicellulose xyloglucan was localized in the zygospore walls of *S. mirabilis* [5]. Nevertheless, the detection of highly complex helicoidal patterns in the endocarp of ‘higher plant’ stone cells in their closest living algal relatives suggests that this trait was inherited from their common algal ancestors and represents another similarity.

### (c) Desiccation-tolerance-enhancing lipid droplet proteins

Studies indicate that the lipid droplets found in streptophyte algae are more similar to those found in land plants than in Chlorophyta [98,99], strengthening the close phylogenetic relationship between the ZCC-grade and Embryophyta. Especially in reproductive structures such as zygospores, spores, seeds and pollen, the trait of lipid droplet accumulation is essential. While they are crucial as an energy and carbon source, particularly after germination, they also play an important role in desiccation tolerance [98,100]. In the latter aspect, the composition of lipid droplet proteins is crucial. Oleosin is a known major lipid droplet coat protein found in seeds [101,102] and pollen, as well as in desiccation-stressed vegetative cells [102,103]. The outer coverage of lipid droplets with this lipid droplet protein is proposed to be a hallmark of desiccation-tolerant tissues [104–106]. Oleosin has also been detected in *Physcomitrium patens* spores [105], a well-studied moss that represents an early divergent land plant lineage. High expression levels of an oleosin homolog were also detected in *Zygnema circumcarinatum* [107], and oleosin proteins were enriched in the lipid droplet proteome of *Mesotaenium endlicherianum* [99]. Furthermore, studies on *Spirogyra grevilleana* showed an increase in oleosin transcript levels during conjugation [108]. As lipid droplet proteins have been shown to differ according to the phylogenetic position, their analysis will provide important insights into the evolutionary history of land plants. While it is not clear whether zygnematophycean lipid droplets are homologous in their molecular makeup to land plants, the detection of oleosin homologs in Zygnematopyhceae and their up-regulation under heat or drought stress [98,99,107,109] suggests that oleosin could be an important lipid droplet protein prior to the evolution of moss spores and seeds. It is, therefore, likely that other features of spores and seeds were co-opted from structures that were present in their common algal ancestor with streptophyte green algae.

## 6. Conclusion

The present review summarizes the current advances in biochemical and ultrastructural investigations on conjugation and zygospore formation in Zygnematophyceae, which is discussed in the context of terrestrialization and land plant evolution. While the conjugation process can be induced only in a few species under laboratory conditions, several new data on sugar residues involved in the conjugation process and the complex cell wall composition of the resulting zygospores became available by CoMPP as well as Raman spectroscopy [5,6,24]. In *Spirogyra pratensis,* even a novel rhamnogalactan protein has been suggested and localized in zygospores and gametangia [7]. The maturation process of zygospores involves reorganizations in storage compounds [9,60], while starch granules are degraded and lipid droplets accumulate, making zygospores a durable structure with enough reserves for germination after a prolonged rest under unfavourable conditions. While zygospore germination has been illustrated in *Mougeotia disjuncta* [6], *Spirogyra mirabilis* [5] and *Zygnema vaginatum* [9], no detailed investigations on the induction of this process are available. Overall, the observed structural and metabolic similarities between present-day streptophyte algae and land plants suggest that the genetic toolkit for many of their defining traits predates the evolution of land plants.

## Data Availability

This article has no additional data.
